# Evaluation of Amino Acids Profile as Non-Invasive Biomarkers of Hepatocellular Carcinoma in Egyptians

**DOI:** 10.3390/tropicalmed7120437

**Published:** 2022-12-13

**Authors:** Samar Ebrahim Ghanem, Mohamed Abdel-Samiee, Hala El-Said, Mohamed I. Youssef, Hassan Ahmed ElZohry, Eman Abdelsameea, Inas Moaz, Sayed F. Abdelwahab, Shymaa A. Elaskary, Eman Mohammed Zaher, Marwa Lotfy Helal

**Affiliations:** 1 Department of Clinical Biochemistry and Molecular Diagnostics, National Liver Institute, Menoufia University, Shebin El-Kom 32511, Egypt; 2 Department of Hepatology and Gastroenterology, National Liver Institute, Menoufia University, Shebin El-Kom 32511, Egypt; 3 Department of Internal Medicine, Faculty of Medicine, Al-Azhar University, Cairo 11651, Egypt; 4 Department of Epidemiology and Preventive Medicine, National Liver Institute, Menoufia University, Shebin El-Kom 32511, Egypt; 5 Department of Pharmaceutics and Industrial Pharmacy, Taif College of Pharmacy, Taif University, P.O. Box 11099, Taif 21944, Saudi Arabia; 6 Department of Medical Microbiology and Immunology, Faculty of Medicine, Menoufia University, Shebin El-Kom 32511, Egypt; 7 Department of Clinical Pathology, Faculty of Medicine, Menoufia University, Shebin El-Kom 32511, Egypt

**Keywords:** aromatic amino acids, branched chain amino acids, BTR, Fischer’s ratio, ultra-performance liquid chromatography, triple quadrupoletandem mass spectrometry

## Abstract

Background: Hepatocellular carcinoma (HCC) is the most dangerous complication of chronic liver disease. It is a multifactorial complicated disease. Hepatitis C and hepatitis B viruses (HCV and HBV, respectively) represent the main causes of HCC in Egypt. Early diagnosis is very important to aid in early intervention. Objectives: The goal of this research is to evaluate the metabolic role of different amino acids as non-invasive biomarkers over the course of HCC. Methods: This study included 302 participants with 97 diagnosed, untreated HCC patients, 81 chronic HCV patients, 56 chronic HBV patients, 18 co-infected patients, and a control group of 50 normal age and gender-matched individuals. All participants provided complete medical histories and underwent complete clinical examinations, abdominal ultrasonography and/or computed tomography, routine laboratory investigations, estimation of serum α-fetoprotein, and determination of amino acid levels using ultra-performance liquid chromatography (UPLC MS/MS). Results: This work revealed a decline in branched chain amino acids (BCAA) and increase in aromatic amino acids (AAA) among infected groups (HCC, HBV, HCV, and co-infected patients) compared to control subjects and a marked change in Fisher’s and the BCAAs/tyrosine molar concentration ratios (BTR) between controls and infected groups. Conclusion: Different amino acids could be used as non-invasive markers to discriminate and follow chronic hepatitis patients to predict the course of HCC.

## 1. Introduction

The liver is the main organ in which most metabolic processes, such as detoxification of blood, production of bile, glucose storage in the form of glycogen, and amino-acid precursor synthesis, occur. 85% of hepatic cells are responsible for these metabolic processes [1]. Thus, it is not surprising that hepatocellular carcinoma (HCC), which is the liver cancer that originates from the hepatic cells, leads to the disturbance of many of the normal metabolic processes to support carcinogenesis [2,3]. Hepatocellular carcinoma plays a major role in mortality around the world. It is the sixth and fourth most prevalent cancer worldwide and in our country, respectively. Epidemiological data of HCC shows variations from place to place. Egypt is the third and fifteenth most populous country in Africa and worldwide, respectively [4]. HCC is a highly malignant tumor that accounts for 85% of total liver cancer cases, usually discovered in the late stages with poor prognosis and few treatment options [5].

The main leading causes of hepatic cancer are hepatitis B and hepatitis C viral (HBV and HCV, respectively) infections and aflatoxin exposure [6]. Other major risk factors include obesity [7], type 2 diabetes, tobacco smoking, and heavy alcohol drinking [5,6]. In contrast, nonalcoholic fatty liver disease (NAFLD) plays a less crucial role in this disease’s process [7]. HBV and HCV infections lead to cirrhosis and metabolic changes, such as those produced by HCC, namely, an increase in long-chain triglycerides and amino acids, such as citrulline and ornithine, which affects the urea cycle [8,9]. Various metabolic disorders related to amino acids, particularly in HCV patients, have been reported [9]. Additionally, HBV/HCV co-infection patients show an increased risk of developing liver damage [10].

Metabolomics yields a global metabolic data analysis for physiological and pathological conditions, considering an organism’s intrinsic properties, such as genetic factors and effects of environment, lifestyle, and diet [11]. Metabolomics are applied in hepatic research and assist in detecting plasma amino acid (AA) disturbances that cause increases in mortality and severity in decompensated cirrhosis cases [12].

The increased dependency of malignancy on some metabolic pathways may cause metabolic changes, which can be targeted by inhibitors of these metabolomics as a treatment of HCC. Thus, numerous metabolomics were studied carefully in HCC to discover their roles in carcinogenesis, but detailed tumorigenic mechanisms have not yet been evaluated [11]. Early screening is important for rapid diagnosis and prognosis. Diagnostic methods for HCC include clinical examination, imaging, and molecular markers and metabolomics analysis [13].

AA metabolism is significantly disturbed in chronic liver disease (CLD) and HCC. These changes lead to an increase in tumor survival, proliferation, and spread [14].

These changes include marked decreases in branched chain amino acids (BCAAs) and increases in aromatic amino acids (AAAs), methionine, and other amino acids. Fischer’s ratio is important for assessing liver metabolism, hepatic functional reserve, and hepatic malformation severity. Fischer’s ratio and the BCAAs/tyrosine molar concentration ratio (BTR) decrease with increasing severity of hepatic damage [12].

Fischer’s ratio is the molar ratio of BCAAs (leucine, valine, and isoleucine) to AAAs (phenylalanine and tyrosine). This value is used for assessment of liver metabolism assessment, liver suppression, and the degree of liver damage [15]. The BTR correlates with numerous liver functions, including hepatic fibrosis markers, blood supply, and liver activity, thus reflecting the degree of hepatic impairment [12]. BTR is used as a HCC prognostic factor, and its value decreases with advanced fibrogenesis in HCV patients’ related chronic liver disease (CLD) [16]. The Fischer’s ratio was employed as a tool to assess the concentration of unbounded amino acids while BTR is a simple form of Fischer’s percentage and is considered as a substitute of Fischer’s ratio as a sign of hepatic damage and reflects chronic liver disease progression [17].

In some studies, the authors reported an increase in certain amino acid levels and a decrease in others. The BTR and Fischer’s ratio differ when comparing HCC patients to healthy participants. The authors deduced that the plasma free amino acids are valuable for early detection and nutritional care in cancer patients. However, few studies have evaluated these changes in Egyptians. Thus, the objective of this research was to check alterations in amino acids metabolism in HCV, HBV, HCV/HBV co-infected, and HCC patients in the Egyptian population.

## 2. Subjects and Methods

### 2.1. Study Population

This case-control study enrolled 302 participants. These included 97 diagnosed, untreated HCC patients (diagnosis was based on clinical evaluation, investigations, ultrasound, and characteristic vascular enhancement patterns detected by multislice triphasic spiral computed tomography [CT] scan or magnetic resonance imaging [MRI]). Another 3 groups were included and consisted of 81 chronically HCV-infected patients, 56 chronically HBV-infected patients, and 18 patients with HCV/HBV co-infection. They presented at the Hepatology and Gastroenterology Outpatient Clinics at the National Liver Institute, Menoufia University from August 2018 to August 2021, in addition to 50 normal age and gender-matched individuals. A written informed consent was obtained from all participants, and the study was approved by the Ethical Committee of National Liver Institute, Menoufia University, Egypt (IRB approval number 00003413). All subjects were clinically assessed. Abdominal and/or CT ultrasound were obtained for all subjects. Laboratory investigations including complete blood count (CBC), liver function tests (aspartate transaminase (AST), alanine transaminase (ALT), bilirubin, albumin, and prothrombin time (PT) and prothrombin concentration (PC)), viral markers (Hepatitis B surface antigen (HBs Ag) and hepatitis C virus antibody (HCV-Ab)), estimation of alpha-fetoprotein (AFP) level, and determination of blood amino acid levels were performed.

### 2.2. Eligibility Criteria

Inclusion criteria:

1. CLD patients with HCV or HBV or co-infection,
2. Confirmed HCC patients with viral infection.

Exclusion criteria:

1. CLD patients due to any causes other than viral infections (such as autoimmune hepatitis, Wilson disease),
2. Other liver tumors (such as hepatoblastoma),
3. Alcohol intake (considering that alcohol intake is very rare in our country).

### 2.3. Sample Collection

Ten milliliters of venous blood were obtained by venipuncture from cubital vein from all participants. Four milliliters were aliquoted into ethyl enediaminetetra acetic acid (EDTA) tubes for CBC and amino acid determination (blood was dripped into a 903 Whatman filter paper and dried), and 3 mL were aliquoted into a sodium citrate tube for PT. The remaining 3 mL were collected in plain tube, left for 10 minutes for clotting, and then centrifuged for 5 minutes at high speed for separation. The collected sera were transferred to 5 mL Eppendorf tubes for hepatic functions, viral diagnosis, and AFP estimation.

### 2.4. Instrument

An ultra-performance liquid chromatography (UPLC) triple quadrupole tandem mass spectrometry (UPLC-TQMS/MS) was used to measure concentrations of AAs from dried blood spots (ACQUITY UPLC H-Class; Waters Corporation, Milford, MA, USA) with a positive electrospray ionization mode, utilizing Mass-Chromosy stems amino acids and Acylcarnitines non-derivatized kit from dried blood (Chromsystems Instruments & Chemicals GmbH, München, Germany). This method uses stable, isotopically-labeled internal standards for calibration and measurement.

### 2.5. Sample Preparation

A 3-mm dried blood spot disk was punched out of the filter card into a microtiter plate well. Thereafter, 100 μL of the reconstituted internal standard was added. The microtiter plate was sealed with a protective sheet and agitated at 600 rpm for 20 min at ambient temperature. The protective sheet was then removed from the plate, and floating liquid was transmitted into a new microtiter plate. The microtiter plate was closed with an aluminum foil for protection. Ten microliters of eluate were injected into the LC–MS/MS system. Additional controls in each analytical run were added for monitoring the precision and accuracy of the analyses.

### 2.6. Chromatography Conditions and Instrument Parameters

The instrument was a UPLC–MS/MS and Masslynx V4.1 software (Waters Corporations, Milford, MA, USA). The MS/MS system contains two mass spectrometers connected in series. In the first mass spectrometer (MS1), the ions were separated based on their mass-to-charge ratio. Subsequently, the ions reached a collision cell in which they dissociated into fragments induced by colliding with an inert gas (argon or nitrogen). After this process, the second mass spectrometer (MS2) analyzed the characteristic fragmentation again based on their mass-to-charge ratio.

### 2.7. Instrument Settings

Ten microliters of the sample were injected into an LC–MS/MS system in which the mobile phase (provided by the kit) was set at 200 µL/min, that was decreased to 20 µL/mL for 0.25 min and increased again to 600 µL/mL for 1.25 min, after which it was decreased to 200 µL/min. The period of use of MS/MS is defined at 1.25 min. The spectra of all analytes were analyzed using the multiple reactions monitoring (MRM) mode. Quantitative analysis was obtained using Neolynx software (Neolynx Inc., Glendale, CA, USA) by comparing the intensity the signal of an analyte versus the corresponding internal standard.

### 2.8. Statistical Methods

Data were taken away and dissected by the statistical package for social science (SPSS) software version 22.0 (IBM Corp Released 2011. IBM SPSS Statistics for Windows, Version 22.0, Armonk, NY, USA) for statistical significance of differences and estimation of the variances among different groups. The Kruskal–Wallis test with post hoc least significant difference correction was utilized for multiple comparisons between quantitative data. The χ2 test was applied to compare the sex ratio between multiple groups. A *p*-value of <0.05 was considered a statistically significant difference. Receiver operating characteristic (ROC) curves were generated, and the corresponding area under the curve (AUC) was calculated. Principal component analysis (PCA) was used to test the discrimination capability of metabolomics models between the studied groups. PCA is a linear transformation that converts original variables (metabolites) into new variables. The results are presented as a scatterplot with each dot on the plot being a sample, while the axes represent the principal components (PCs).

## 3. Results

### 3.1. Study Population’s Demographic and Basic Biochemical Data

As shown in Table 1, the demographic criteria of the enrolled subjects in the present study were well matched in age and gender (*p* > 0.05). A highly significant decline in white blood cells (WBCs), international normalized ratio (INR), prothrombin time (PT), platelets, and albumin levels (*p* < 0.001) and an increase in hemoglobin (HB), bilirubin, creatinine, and AFP levels (*p* < 0.001) were found in HCC patients.

As shown in Table 2, significant variations between different biochemical parameters and different amino acids were detected (*p* < 0.05).

### 3.2. Comparison of Blood Amino Acids Levels in Study Groups

There were highly statistically significant differences among study groups in terms of aromatic and branched chain amino acids, BTR, and Fisher’s ratio (*p* < 0.001), as shown in Table 3.

Importantly, the current study showed a highly statistically significant difference between control subjects and HCC groups (*p* < 0.001)with an increase in aspartate, citrulline, glutamate, proline, glycine, phenylalanine, tyrosine, ornithine, methionine, glycine/alanine ratio, and arginine (aromatic amino acids), a decrease in valine and leucine isoleucine ratio (branched chain) in HCC patients, and a significant increase (*p* = 0.02) in the leucine/phenylalanine ratio, with no differences in other amino acid levels (Table 3).

**Figure 1:**
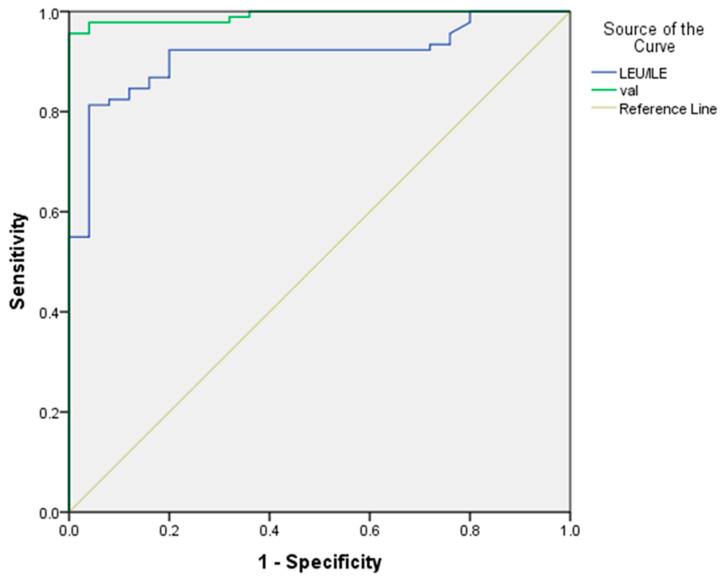
Receiver operating characteristic curves for the BCAA to discriminate between HCC and control, the AUC for Leucine/Isoleucine was 0.91(95%CI = 0.86–0.96). At cut off point = 174.0, The sensitivity was 87.0%, and specificity was 80.0%. For Valine the AUC was 0.99 (95%CI = 0.98–0.100). At cut off point = 150.3, the sensitivity was 98.0%, and specificity was 88.0%; AUC, area under curve.

BCAA can be used to differentiate between two groups (HCC and control) based on the ROC curve for which the area under the curve (AUC) was 0.912 for leucine and isoleucine with asensitivity of 87%. For valine, when the AUC was 0.992, the sensitivity was 98% (Figure 1).

Highly statistically significant variations between control and HBV groups were detected (*p* ≤ 0.001) with an increase incitrulline/phenylalanine ratio, glycine, glycine/alanine ratio, leucine/alanine ratio, and an increase (*p* < 0.002) in citrulline and a decrease in valine, alanine, and the leucine/isoleucine ratio in HBV, with no statistically significant difference in other amino acid levels (Table 3).

A highly statistically significant variation was observed between control and HCV subjects with an increase in aspartate, citrulline, proline, citrulline phenylalanine ratio, glutamate, glycine, glycine alanine ratio, ornithine, phenylalanine (*p* ≤ 0.001), and tyrosine (*p* = 0.02) and a decrease in valine, alanine, leucine isoleucine ratio, and citrulline phenylalanine ratio in HCV, with no statistically significant difference in other amino acid levels (Table 3).

A highly statistically significant variation was detected between co-infection and control subjects (*p* ≤ 0.001) with an increase in glutamate, glycine, aspartate, and leucine/alanine ratio and a significant difference (*p* < 0.05) in citrulline, proline, arginine, and glycine/alanine ratio. A decrease in the leucine/isoleucine ratio and valine in co-infection with no statistically significant difference in other amino acid levels was found (Table 3).

Comparisons between HCC and HBV groups showed a highly significant increasein aspartate, glutamate, methionine, valine, alanine, and the citrulline/phenylalanine ratio (*p* ≤ 0.001), a significant increase (*p* < 0.01) in arginine and tyrosine, and a significant increase in phenylalanine (P5 = 0.007), with no statistically significant difference in other amino acid levels (Table 3).

**Figure 2:**
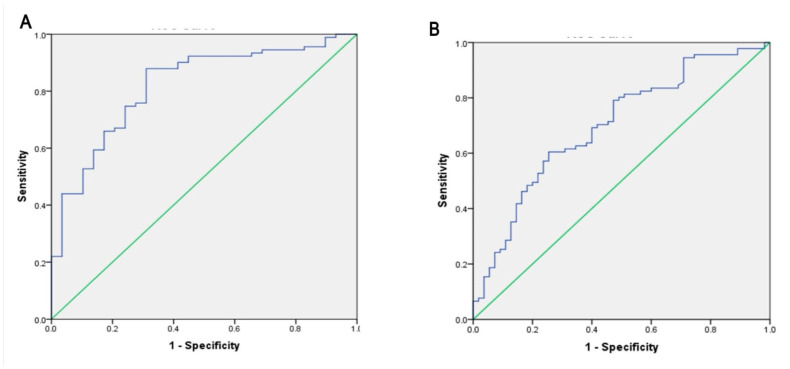
Receiver operating characteristic curve for amino acids Between HCC and HBV. (**A**) For Phenylalanine, tyrosine, phy:tyr (AAA). The AUC was 0.81(95%CI = 0.73–0.90). (**B**) for Urea cycle metabolites (Aspartate, arginine, citrulline, ornithine). The AUC was 0.76 (95%CI = 0.65–0.86).

AAA (tyrosine and phenylalanine) were useful in differentiating between two groups based on the ROC curve for which the AUC was 0.81 and based on urea cycle metabolites (aspartate, arginine, citrulline, ornithine) (Figure 2A) & for which the AUC was 0.76 as shown in Figure 2B. 

A highly significant difference was observed between HCC and HCV patients (*p* ≤ 0.001) with an increase in methionine and alanine and a statistically significant difference (*p* < 0.05) in the methionine/phenylalanine, phenylalanine/tyrosine, and citrulline/phenylalanine ratios, with no statistically significant difference in other amino acid levels (Table 3).

**Figure 3:**
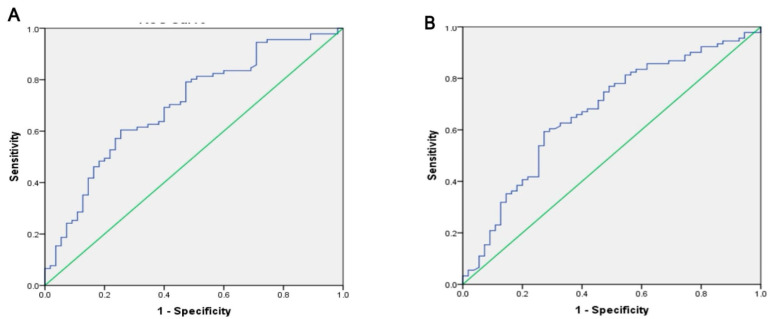
Receiver operating characteristic curve for amino acids Between HCC and HCV. (**A**) For Phenylalanine, tyrosine, phy:tyr (AAA). The AUC was 0.69 (95%CI = 0.61–0.78). (**B**) for Urea cycle metabolites (Aspartate, arginine, citrulline, ornithine). The AUC was 0.67 (95%CI = 0.58–0.76).

AAAs were also useful in differentiating between HCC and HCV based on a ROC curve, for which the area under the curve was 0.699 (Figure 3A), and by urea cycle metabolites, where the AUC was 0.667, as shown in Figure 3B. 

A highly statistically significant difference was observed between HCC and the co-infected groups (*p* = 0.001), with a significant increase in methionine and glycine/alanine ratio (P7 = 0.04) and a decrease in the leucine/isoleucine ratio in HCC, with no significant difference regarding other amino acid levels (Table 3).

Significant variations between HBV and HCV groups with an increase in glutamate, phenylalanine, and tyrosine (*p* < 0.005) and ornithine (*p* = 0.006) in HCV patients with no statistically significant difference in other amino acids (Table 2).

A highly statistically significant difference was observed between HBV and co-infected patients (*p* ≤ 0.001), with an increase in glutamate, valine and alanine, andthe citrulline/phenylalanine ratio, and a significant difference (*p* < 0.05) in aspartate and arginine was found with no difference in other amino acid levels (Table 3).

Furthermore, highly statistically significant differences between HCV and co-infected patients (*p* ≤ 0.001) with a significant increase in valine and alanineand a significant difference (*p* < 0.05) in the citrulline/phenylalanine ratio andglycine/alanine ratiowere found, with no significant difference in other amino acid levels (Table 3).

Regarding Fischer’s ratio, this study showed a highly significant decrease (*p* < 0.001) between the control and HCC, control and HBV, control and HCV, control and coinfected study groups, and HCC and HBV subjects, with a marked decrease in HCC patients (*p* = 0.02 between HBV and HCV) (as shown in Table 3).

Regarding BTR, a highly significant decrease was observed (*p* < 0.001) between the patient control and HCC groups, control and HBV groups, control and HCV groups, and control and co-infected patients, with a marked decrease in the HCC group (Table 3).

**Figure 4:**
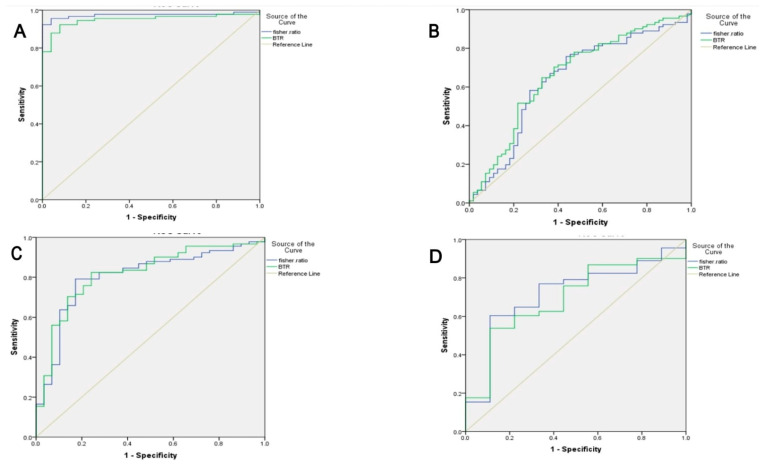
Receiver operating characteristic curve for Fisher ratio and BTR Between studied groups. (**A**) Between HCC and Controls, AUC for fisher ratio was 0.97. For BTR the AUC was 0.95. (**B**) Between HCC and HCV. AUC for fisher ratio was 0.64. For BTR the AUC was 0.67. (**C**) Between HCC and HBV, AUC for fisher ratio was 0.80. For BTR the AUC was 0.82. (**D**) Between HCC and Co-infection, AUC for fisher ratio was 0.72. For BTR the AUC was 0.69.

After applying the ROC curve analysis for the Fisher ratio, it was found that AUC was 97.4, sensitivity 96.7%, and specificity was 88% between HCC and control. After applying the ROC curve analysis for BTR, it was found that AUC was 95, sensitivity 94.5%, and specificity was 84% between HCC and control (as shown in Figure 4A).

After applying a ROC curve analysis for the Fisher ratio, it was found that the AUC was 64.2, sensitivity 67%, and specificity was 64% between HCC and HCV. After applying the ROC curve analysis for BTR, it was found that the AUC was 66.5, sensitivity was 65.9%, and specificity was 64% between HCC and HCV (Figure 4B).

Using ROC curve analysis for Fisher, the AUC was 80.4, sensitivity was 82.4%, and specificity was 73% between HCC and HBV. Using ROC curve analysis for BTR, AUC was 81.7, sensitivity was 82.4%, and specificity was 76% between HCC and HBV (Figure 4C).

Using ROC curve analysis for the Fischer ratio, AUC was 71.8, sensitivity was 76.9%, and specificity was 67% between HCC and co-infection. Using ROC curve analysis for BTR, AUC was 69.4, sensitivity was 62.6%, and specificity was 67% between HCC and co-infection (Figure 4D).

**Figure 5:**
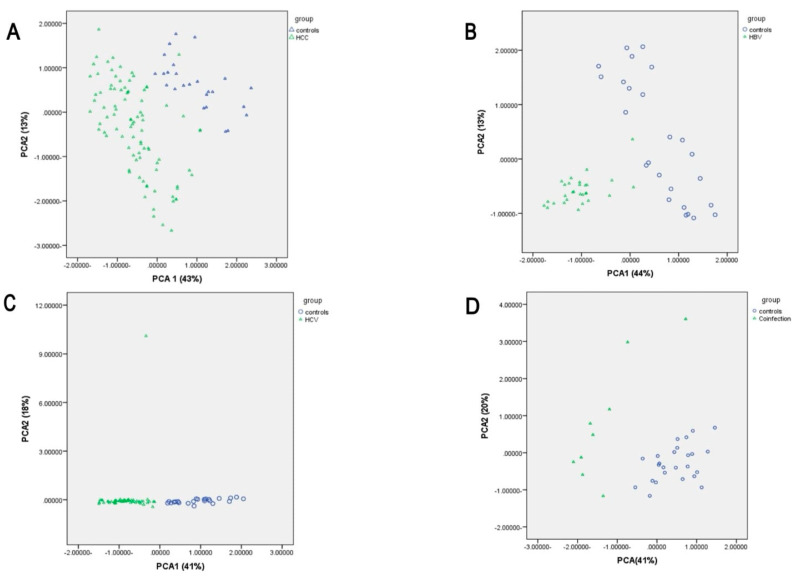
Principle component analysis (PCA) for amino acids Between studied groups. (**A**) Between HCC and Controls. (**B**) Between HBV and controls. (**C**) Between HCV and controls. (**D**) Between Co-infection and controls AUC for fisher ratio was 0.72.

After applying PCA designs, which relied on the UPLC analysis of amino acid markers, good discrimination between HCC and control groups by nine amino acids markers and three ratios with good separation of the samples in two different places was found, and AUC was 0.941 (Figure 5A).

Good discrimination between HBV and control groups based on nine amino acid markers and two ratios with good separation of the samples in two different places was found, and the area AUC was 0.975 (Figure 5B).

Good discrimination between HBV and control groups based on nine amino acid markers and two ratios with good separation of the samples in two different places was found, and the area AUC was 0.975 (Figure 5B).

Good discrimination between HCV and control groups based on seven amino acid markers and four ratios with good separation of the samples in two different places was found, and the AUC was 0.985 (Figure 5C).

Good discrimination between co-infected and control groups based on seven amino acid markers and two ratios with good separation of the samples in two different places was noted, and the AUC was 0.933 (Figure 5D).

A small separation between HCC and HCV groups based on seven amino acid markers, including BCAA, and one ratio with moderate scattering of the samples in two different places was found, and the AUC was 0.567 (Figure 5D).

## 4. Discussion

Globally, HCC is the third most common cause of cancer-related deaths. Clinical manifestations mostly appear in late stages when treatment becomes difficult. Therefore, guidelines advise frequent follow-up for rapid screening of a tumor [13]. Metabolomics is a promising non-invasive tool for early detection of pathological alterations in HCC patients. Importantly, most HCC patients were found to have disturbances in protein metabolism [18,19].

The complexity of protein metabolic changes in cancer patients may be presented with alterations in plasma free amino acids [19]. In the current study, we noticed several amino acid alterations among the study groups (HCC, HBV, HCV, co-infected, and control). Our findings reported elevations in all amino acids in all study groups except BCAA (leucine, isoleucine, and valine), that decreased in all patient groups compared to the control group. These results agree with the findings of other HCC metabolomic reports; another study found an increase in serine, glycine, aspartate, glutamate, and phenylalanine in cirrhotic patients versus healthy controls, with consistent elevations in HCC [19]. Another report examined the role of elevatedions in different amino acids of alanine, aspartate, glutamate, glycine, serine, and threonine in metabolic changes in HCC patients, and they found that the amino acid metabolites, including L-serin and glycine, were elevated in HCC patients compared to control subjects. The authors attributed this finding to impaired liver function, as many enzyme systems are damaged and a vast number of amino acids are released from the liver, leading to an increase in their concentrations in the blood [20].

Furthermore, amino acid disturbance in the serum of HCC and cirrhotic patients were reported, in which BCAAs were found to decrease and AAAs increased, particularly tyrosine, due to deregulation of tyrosine, phenylalanine, and tryptophan metabolism and biosynthesis. BCAA degradation and biosynthesis were also disrupted. These findings support the pathological progression of HCC, including an initial increase in the biosynthesis of BCAAs and AAAs and eventual increase in BCAAs degradation [21,22]

In study by Gao et al. [23], it was reported that the increased levels of AAA (phenylalanine and tyrosine) were associated with liver cirrhosis and HCC. Jain et al., in 2012, explained the crucial role of glycine in purine synthesis of the malignant tumor cells [24]. Amelio et al.,2014confirmed the important function of glycine in cancer development and progression [25], and Nilssonaetal. also demonstrated that elevatedGlycine levels in HCC are an essential componentof proliferating cancer cells [26].

Importantly, a report supported findings ofhigh levels of amino acids among HCC patients compared to chronic liver disease patients and attributed this to cancer related enzymes, such as enzymes of glycolysis, serine proteases, and phosphoenol pyruvate carboxylase [27]. Moreover, one study used isoleucine (AAA) as a biomarker to distinguish between HCC and healthy controls and assumed that the metabolic signature associated with HCC occurrence presented higher levels of AAAs and lower levels of BCAA when compared to the healthy subjects [28]. In this regard, one report found an increase in plasma AAAs and methionine levels in cirrhotic patients without HCC, but these were not obvious in cirrhotic patients with HCC [29].

Higher serum methionine concentrations in patients infected with HCV were found compared to HCC patients. This evidence agrees with the finding that deregulated lipid metabolism is related to HCC development [30]. In addition, Chen et al. observed lower levels of the urea cycle metabolitescitrulline, arginine, and ornithine in HCC patients compared to healthy controls, and decreased levels were associated with advanced tumor stages [31].

### 4.1. Amino Acids in HBV

In the current study, we found an increase in the level of most amino acids in HBV patients. In this regard, one study also reported metabolic changes in essential amino acids during different stages of HBV infection [32]. Likewise, another reportrevealed increases in levels of serine, alanine, glycine, cysteine, aspartic acid, methionine, tyrosine, tryptophan, and phenylalanine in HBV-related HCC, which may be explained by the rapid proliferation of tumor cells and urea cycle metabolites (aspartate, arginine, citrulline, and ornithine) in HBV infection as a marker explaining deterioration of liver status [8]. They also reportedthat most amino acids (glycine, phenylalanine, glutamic acid, asparagine, and tyrosine) were elevated in HBV infection compared to controls [8,32]. Another study found that urea cycle metabolites increased in HBV infection and explained that this finding as due to an HBV attack on a glucose triphosphate–nicotinic adenine dinucleotide (NADH) shuttle and a decrease in the citrin carrier, which impairs aspartate and glutamate in normal liver tissue. Aspartate combines with citrulline to form arginino-succinate, which yields arginine that is converted to ornithine, is transported across mitochondrial membranes, and leads to elevation of aspartate, citrulline, and glutamate as a result of permanent destruction of liver cells by chronic viral infection [21].

In another study by Wu et al., it was observed that HCC patients have more significant decreases in levels of leucine, lysine, threonine, tryptophan, and valine than in chronic hepatitis B (CHB) patients. In contrast, the level of phenylalanine, one of the AAAs, was significantly increased. Scatter diagrams showed differences in these amino acids between CHB and HCC patients and proposed that the increase in phenylalanine level due to seriously hepatic impairment in HCC patients versus CHB patients because of phenylalanine is mainly metabolized in the liver [33].

### 4.2. Amino Acids in HCV

We found a significant increase in AAAs and urea cycle metabolites (aspartate, arginine, citrulline, and ornithine) in HCV patients. HCV was found to induce significant changes in levels of several amino acids (alanine, valine, tryptophan, and isoleucine) [34]. In this regard, one study found that most amino acids levels were elevated in a persistently HCV-infected cell line. As that cells showed, prominent steatosis supported prolonged HCV infection for more than two years, and the citric acid cycle was preferably facilitated over the glycolysis pathway, with a prominent rise in most amino acids [35]. In addition, a recent study showed that HCV patients had higher serum methionine concentrations compared to the HCC patients. Methionine metabolism was closely related to diverse pathophysiological processes. Therefore, changes in the methionine cycle play a pathogenic role in CLD [36]. Similarly, another study found statistically significant differences between HCC and HCV patients in AAAs and urea cycle metabolites [37]. Additionally, another report showed that disturbance of different amino acids with more deterioration in liver function occurred in HBV and HCV co-infection [30]. In this study, a highly significant decrease in Fisher’s and BTR ratios (*p* < 0.001) occurred in the studied patient groups (HCC, HBV, HCV, and co-infection) compared to the control subjects. In this regard, a recent report showed that a low Fisher’s ratio correlated with the reduction in synthetic function of the liver, mainly albumin, and considered it as a good prognostic factor of liver disease [38].

It was assumed that fluctuations in free amino acid plasma levels would be particularly found in compensated and uncompensated cirrhosis. Therefore, amino acid metabolic alterations in the liver become more dangerous as the state of chronic liver disease worsens and include marked decreases in BCAAs and increases in AAAs, methionine, and other amino acids. Further, Fischer’s ratio and BTR decrease with increasing hepatic damage severity [15,16]. In addition, BTR decreased in cirrhotic patients, and BTR is widely used in Japan as an easily measurable index of amino acid disturbance [39,40].

The current study showed disturbances in AAA and BCAA biosynthesis, metabolism, and degradation. In the same line, a recent study recognized amino acid imbalance in HCC and chronic hepatitis C patients, showing lower levels of BCAAs and higher levels of AAAs, particularly tyrosine [17]. These findings support the pathologic course of HCC. At first, increased BCAAs biosynthesis and AAAs, and finally elevated degradation of BCAAs, partly reflecting elevated serum carnitine level, which oxidizes BCAAs [41,42]. This is supported by a metabolomic study which found that phenylalanine increases in HCC patients’ sera compared to healthy controls and explained that decreased serum BCAAs may also correlate with elevated serum carnitine levels, as they have the ability to oxidize BCAAs [17].

Importantly, the decrease in BCAA and the increase in AAA were used to differentiate between HCC and healthy controls [42]. Additionally, one report discriminated between CLD and healthy controls based on reduced amino acids levels in BCAAs and the increase in AAAs, especially tyrosine [43]. BCAA metabolic pathways reflect liver responses to disease, and these pathways play an essential role in tumor development and progression [22,41]. Other studies [39,43] supported our results in that they clarified the changes in amino acid metabolism in chronic liver diseases in the form of a disturbance in the levels of AAAs or BCAAs, reflecting that a hormonal imbalance (insulin/glucagon) and a reduction in liver functions might be the main causes.

## 5. Conclusions

An observed difference in some specific blood amino acids, Fisher ratio, and BTR was found among HCC and another patient group, which may be used to anticipate HCC development during the early stages, although further studies are needed to confirm these changes.

Amino acids, especially BCAA and AAA, were found to be unconventional variables related to the occurrence of HCC, and screening of amino acids could be used in clinical practice to detect new cases of HCC in high-risk groups. Moreover, we emphasize that BTR and Fisher’s ratio are important in monitoring the liver synthetic status.

## Figures and Tables

**Table 1:** Basic and biochemical characteristics of the studied groups.

	Controls (n = 50)	HCC (n = 97)	HBV (n = 56)	HCV (n = 81)	Coinfection (n = 18)	*p* Value	Post Hoc Test
Age Mean ± SD	57.62 ± 6.05	59.44 ± 7.6	57.05 ± 6.04	58.06 ± 7.85	56.78 ± 4.6	0.23	
Gender Male Female	45(90.0%) 5 (10.0%)	83 (85.6%) 14 (14.4%)	48 ( 85.7%) 8(14.3%)	69 (85.2) 12 (14.8%)	16 (88.9%) 2 (11.1%)	0.93	
HB (g/dl)	12.25 (11.8–12.9)	13.6 (12.5–14.8)	12.0 (11.42–13.2)	12.0 (11.9–13.2)	12.0 (11.9–13.2)	<0.001	P1 = 0.001, P2 = 0.99, P3 = <0.001, P4 = 0.99, P5 = 0.001, P6 = 0.21, P7 = 0.13, P8 = <0.001, P9 = 1.0, P10 = 0.003.
WBCs (10^9^/L)	4.0 (3.8–4.2)	5.4 (4.4–7.0)	6.0 (4.72–7.0)	6.2 (4.75–7.35)	6.2 (4.75–7.35)	<0.001	P1 = <0.001, P2 = <0.001, P3 = <0.001, P4 = 0.002, P5 = 0.98, P6 = <0.001, P7 = 0.99, P8 = 0.006, P9 = 1.00, P10 = 0.33.
Platelets (10^9^/L)	378.0 (340–401)	142.0 (102.5–171.0)	209.5 (184.0–248.7)	204.0 (147.5–223.7)	204.0 (147.5–223.7)	<0.001	P1 = <0.001, P2 = <0.001, P3 = <0.001, P4 = <0.001, P5 = <0.001, P6 = <0.001, P7 = 0.05, P8 = 0.60, P9 = 0.89, P10 = 0.17.
ALT (IU/L)	25.0 (18.0–30.0)	52.0 (42.0–64.0)	39.5 (20.5–64.0)	56.0(37.7–62.0)	56.0 (37.7–62.0)	<0.001	P1 = <0.001, P2 = 0.06, P3 = <0.001, P4 = 0.001, P5 = 1.0, P6 = 0.79, P7 = 0.61, P8 = 0.99, P9 = 0.99, P10 = 1.0.
AST (IU/L)	25.0 (22.0–28.2)	56.0 (44.5–71.0)	35.0 (24.0–57.75)	57.0 (40.7–67.0)	57.0 (40.7–67.0)	<0.001	P1 = <0.001, P2 = 0.008, P3 = <0.001, P4 = <0.001, P5 = 0.26, P6 = 0.01, P7 = 0.72, P8 = 1.0, P9 = 0.99, P10 = 0.95.
Albumin (g/dL)	4.10 (4.20–4.42)	3.7 (3.05–4.0)	4.1 (4.0–4.3)	4.0 (3.9–4.15)	4.0 (3.9–4.15)	<0.001	P1 = <0.001, P2 = 0.57, P3 = 0.95, P4 = 0.33, P5 = <0.001, P6 = <0.001, P7 = 0.001, P8 = 0.99, P9 = 0.98, P10 = 0.74,
Bilirubin total (mg/dl)	0.80 (0.65–0.82)	0.90 (0.69–1.2)	0.8 (0.63–1.0)	0.75 (0.70–0.82)	0.75 (0.70–0.82)	<0.001	P1 = <0.001, P2 = 0.37, P3 = 0.008, P4 = 0.69, P5 = 0.81, P6 = <0.001, P7 = 0.29, P8 = 0.008, P9 = 1.0, P10 = 0.02,
Bilirubin D (mg/dl)	0.20 (0.20–0.30)	0.34 (0.20–0.60)	0.30 (0.28–0.60)	0.35 (0.30–0.55)	0.35 (0.30–0.55)	<0.001	P1 = <0.001, P2 = 0.005, P3 = 1.0, P4 = 0.005, P5 = 1.0, P6 = <0.001, P7 = 1.0, P8 = 0.004, P9 = 0.99, P10 = 0.004,
PT (minutes)	91.0 (89.0–93.0)	82.0 (73.0–88.5)	83.5 (79.2–90.0)	84.0 (77.5–90.5)	84.0 (77.5–90.5)	<0.001	P1 = <0.001, P2 = <0.001, P3 = 1.0, P4 = 0.01, P5 = 0.80, P6 = <0.001, P7 = 0.99, P8 = <0.001, P9 = 1.0, P10 = 0.007,
INR	2.9 (2.8–3.1)	1.14 (1.10–1.26)	1.2 (1.1–1.2)	1.16 (1.09–1.22)	1.16 (1.09–1.22)	<0.001	P1 = <0.001, P2 = <0.001, P3 = <0.001, P4 = <0.001, P5 = 0.99, P6 = <0.001, P7 = 1.0, P8 = <0.001, P9 = 1.0, P10 = 0.01,
Creatinine (mg/dL)	0.9 (0.8–0.9)	0.90 (0.79–1.0)	0.8 (0.70–0.90)	0.72 (0.61–0.82)	0.80 (0.70–0.86)	<0.001	P1 = 0.29, P2 = 0.25, P3 = <0.001, P4 = 0.98, P5 = 0.01, P6 = <0.001, P7 = 0.45, P8 = 0.12, P9 = 1.0, P10 = 0.48,
AFP (ng/mL)	1.10 (0.80–1.55)	75.95 (11.24–272.5)	3.3 (2.7–5.0)	3.0 (2.1–6.05)	3.1 (2.75–6.74)	<0.001	P1 = 0.27, P2 = <0.001, P3 = 0.03, P4 = 0.001, P5 = 0.03, P6 = 0.02, P7 = 0.16, P8 = 0.98, P9 = 1.0, P10 = 0.99

Kruskal-Wallis was used for comparison between ≥ groups for not normally distributed quantitativevariable. P1 = control and HCC, P2 = control and HBV, P3 = control and HCV, P4 = control and coinfection, P5 = HCC and HBV, P6 = HCC and HCV, P7 = HCC and Coinfection, P8 = HBV and HCV, P9 = HBV and Coinfection, P10 = HCV and Coinfection.

**Table 2:** Correlation between biochemical parameters and all amino acids.

Studied variables	ALT (IU/L)	AST (IU/L)	Albumin (g/dL)	Bilirubin T (mg/dl)	Bilirubin D (mg/dl)	PT%	INR	Creatinine (mg/dl)	AFP (ng/mL)	Child Score
Aspartate (μmol/L)	*−0.167	*−0.171	0.068	−0.083	−0.094	0.079	*0.283	0.009	*−0.206	*−0.563
ctr: phe	*−0.142	*−0.175	0.048	0.037	−0.005	0.101	*0.335	*0.156	*−0.221	*−0.510
Citrulline (μmol/L)	0.127	*0.210	−0.115	0.076	*0.135	−0.110	*−0.286	0.092	*0.277	*0.430
Glutamate (μmol/L)	−0.079	−0.018	0.039	−0.019	−0.016	0.101	*0.201	0.010	−0.104	*−0.304
Glycine (μmol/L)	*−0.209	*−0.144	0.027	0.002	0.022	−0.043	*0.189	0.018	−0.122	*−0.180
Gly/ALA	0.041	0.049	*−0.137	0.027	−0.060	*−0.131	−0.106	−0.055	*0.166	*0.292
LEU/ILE	*−0.267	*−0.301	*0.362	*−0.151	*−0.157	*0.239	*0.309	0.024	*−0.401	*−0.681
LUE/ALA	0.027	−0.032	*0.147	*−0.144	−0.107	0.081	−0.123	−0.057	−0.030	−0.060
LUE/PHE	*−0.175	*−0.213	*0.319	*−0.188	−0.114	*0.258	−0.064	−0.099	*−0.232	*−0.317
Methionine (μmol/L)	−0.089	−0.100	−0.034	0.127	0.001	−0.038	*0.520	*0.241	*−0.161	*−0.495
met:ph	−0.129	*−0.154	−0.006	0.085	−0.057	0.016	*0.514	*0.231	*−0.196	*−0.620
ornithine	−0.079	−0.050	0.036	−0.038	−0.054	0.036	*0.197	0.057	*−0.148	*−0.254
Proline (μmol/L)	*−0.162	*−0.178	*0.179	−0.048	*−0.177	0.054	*0.263	0.067	*−0.274	*−0.444
phenylalanine(μmol/L)	*0.176	*0.275	*−0.136	0.116	0.121	*−0.201	*−0.224	−0.040	*0.310	*0.403
ph:tyr	*−0.175	*−0.182	*0.152	*−0.153	−0.093	*0.171	−0.124	−0.037	*−0.226	*−0.201
Tyrosine (μmol/L)	*0.243	*0.295	*−0.212	*0.181	*0.147	*−0.267	−0.022	0.035	*0.338	*0.354
Valine (μmol/L)	*−0.191	*−0.216	*0.171	−0.036	−0.073	*0.161	*0.378	0.040	*−0.299	*−0.786
alanine (μmol/L)	*−0.163	−0.103	0.059	*0.114	0.066	0.013	*0.367	0.074	*−0.196	*−0.524
arginine (μmol/L)	*−0.145	−0.107	0.053	0.042	−0.008	0.030	*0.426	0.086	*−0.277	*−0.657
Fischer‘s ratio	*−0.421	*−0.497	*0.422	*−0.229	*−0.241	*0.399	*0.331	0.010	*−0.610	*−0.749
BTR	*−0.423	*−0.480	*0.407	*−0.250	*−0.244	*0.393	*0.268	−0.017	*−0.583	*−0.711

* *p* = 0 < 0.05.

**Table 3:** Comparison of blood amino acids levels in studied groups (μmol/l):.

	Controls (n = 50)	HCC (n = 97)	HBV (n = 56)	HCV (n = 81)	Coinfection (n = 18)	*p* Value	Post Hoc Test
ASP	52.1 (40.4–56.2)	109.0 (58.5–164.5)	64.9 (0.0–136.75)	121.0 (0.0–200.5)	160.0 (97.92–226.25)	<0.001	P1 = <0.001, P2 = 0.40, P3 = <0.001, P4 = 0.001, P5 = 0.005, P6 = 1.00, P7 = 0.67, P8 = 0.06, P9 = 0.01, P10 = 0.51
Cit. phe	0.35 (0.28–0.43)	0.30 (0.21–0.40)	0.18 (0.0–0.36)	0.24 (0.0–0.36)	0.30 (0.28–0.33)	<0.001	P1 = 0.15, P2 = <0.001, P3 = <0.001, P4 = 0.57, P5 = <0.001, P6 = 0.01, P7 = 1.00, P8 = 0.88, P9 = 0.001, P10 = 0.02
Citruline	18.2 (14.15–24.7)	30.6 (22.9–36.8)	27.9 (20.5–35.13)	31.6 (25.33–39.0)	27.6 (22.3–37.6)	<0.001	P1 = <0.001, P2 = 0.002, P3 = <0.001, P4 = 0.01, P5 = 0.99, P6 = 0.80, P7 = 0.99, P8 = 0.53, P9 = 0.96, P10 = 1.0
Glu	95.7 (76.97–126.7)	180.0 (135.5–242.5)	110.5 (0.0–170.2)	194.0 (0.0–288.5)	194.0 (171.7–224.25)	<0.001	P1 = <0.001, P2 = 0.99, P3 = <0.001, P4 = <0.001, P5 = <0.001, P6 = 0.93, P7 = 0.98, P8 = 0.001, P9 = <0.001, P10 = 0.59
Gly	97.7 (76.02–125.0)	175.0 (150.0–203.0)	166.0 (154.0–195.0)	174.0 (155.0–210.0)	180.0 (143.0–203.5)	<0.001	P1 = <0.001, P2 = <0.001, P3 = <0.001, P4 = <0.001, P5 = 1.0, P6 = 1.0, P7 = 0.99, P8 = 1.0, P9 = 1.0, P10 = 0.99
Gly/ALA	0.59 (0.54–0.73)	0.95 (0.80–1.09)	0.98 (0.81–1.18)	0.96 (0.83–1.13)	0.80 (0.71–0.93)	<0.001	P1 = <0.001, P2 = <0.001, P3 = <0.001, P4 = 0.01, P5 = 0.99, P6 = 1.0, P7 = 0.04, P8 = 1.0, P9 = 0.05, P10 = 0.03
Leu. Ile	187.4 (175.15–198.9)	121.0 (93.0–140.0)	119.0 (101.0–145.0)	127.0 (108.0–151.0)	140.0 (130.7–153.5)	<0.001	P1 = <0.001, P2 = <0.001, P3 = <0.001, P4 = <0.001, P5 = 1.0, P6 = 0.74, P7 = 0.04, P8 = 0.99, P9 = 0.32, P10 = 0.55
LUE/ALA	0.36 (0.28–0.40)	0.42 (0.38–0.50)	0.46 (0.42–0.54)	0.47 (0.40–0.55)	0.50 (0.43–0.54)	<0.001	P1 = 0.82, P2 = <0.001, P3 = 0.96, P4 = <0.001, P5 = 0.87, P6 = 1.0, P7 = 0.87, P8 = 0.97, P9 = 1.0, P10 = 0.98
LUE/PHE	1.56 (1.43–1.82)	1.40 (1.15–1.68)	1.84 (1.51–2.07)	1.58 (1.29–1.81)	1.68 (1.58–1.79)	<0.001	P1 = 0.02, P2 = 0.36, P3 = 0.98, P4 = 0.99, P5 = 0.001, P6 = 0.96, P7 = 0.13, P8 = 0.99, P9 = 0.99, P10 = 0.99
Met	5.15 (4.4–6.3)	9.09 (6.73–11.9)	5.18 (4.26–6.81)	2.91 (4.4–7.09)	5.65 (5.1–7.6)	<0.001	P1 = <0.001, P2 = 0.99, P3 = 0.99, P4 = 0.67, P5 = <0.001, P6 = <0.001, P7 = 0.001, P8 = 1.0, P9 = 0.98, P10 = 0.99
Met/Phe	0.11 (0.09–0.13)	0.17 (0.12–0.22)	0.11 (0.09–0.14)	0.07 (0.05–0.12)	0.11 (0.09–0.12)	<0.001	P1 = 0.95, P2 = 0.97, P3 = 0.49, P4 = 0.96, P5 = 0.99, P6 = 0.04, P7 = 0.95, P8 = 0.96, P9 = 0.97, P10 = 0.99
Orn	100.0 (82.82–112.25)	119.0 (89.5–149.0)	87.4 (78.4–105.0)	122.0 (109.0–137.0)	128.0 (79.7–148.5)	<0.001	P1 = <0.001, P2 = 1.0, P3 = <0.001, P4 = 0.48, P5 = 0.01, P6 = 1.0, P7 = 0.99, P8 = 0.006, P9 = 0.69, P10 = 0.98
Proline	90.4 (66.85–107.25)	114.0 (91.9–136.0)	82.8 (103.0–29.5)	120.0 (96.5–151.0)	114.0 (96.8–138.5)	<0.001	P1 = <0.001, P2 = 0.20, P3 = <0.001, P4 = 0.04, P5 = 0.63, P6 = 0.98, P7 = 1.0, P8 = 0.21, P9 = 0.97, P10 = 0.98
Phe	49.4 (41.05–50.9)	59.8 (48.5–75.70)	46.4 (42.65–58.97)	60.7 (52.5–71.2)	60.4 (52.2–72.1)	<0.001	P1 = <0.001, P2 = 0.73, P3 = <0.001, P4 = 0.009, P5 = 0.007, P6 = 0.98, P7 = 1.0, P8 = 0.004, P9 = 0.12, P10 = 1.0
Ph/tyr	0.84 (0.8–1.02)	0.79 (0.69–0.94)	1.0 (0.86–1.23)	0.97 (0.81–1.23)	0.90 (0.81–1.19)	<0.001	P1 = 0.38, P2 = 0.19, P3 = 0.31, P4 = 0.99, P5 = 0.01, P6 = 0.004, P7 = 0.24, P8 = 0.99, P9 = 0.74, P10 = 0.97
Tyrosine	57.0 (51.57–63.25)	74.0 (58.0–101.0)	46.5(38.2–59.2)	66.3 (52.2–81.2)	58.0 (46.17- 90.8)	<0.001	P1 = 0.03, P2 = 0.48, P3 = 0.02, P4 = 0.40, P5 = 0.004, P6 = 0.56, P7 = 0.911, P8 = 0.002, P9 = 0.08, P10 = 1.0
Valine	168.1 (157.1–173.2)	86.0 (67.5–108.0)	58.5 (0.0–99.6)	85.9 (0.0–106.5)	95.9 (85.3–101.2)	<0.001	P1 = <0.001, P2 = <0.001, P3 = <0.001, P4 = <0.001, P5 = <0.001, P6 = 0.09, P7 = 0.18, P8 = 0.49, P9 = <0.001, P10 = 0.001
Alanine	197.0 (168.7–229.0)	184.0 (148.0–211.5)	128.0 (0.0–181.0)	166.0 (0.0–201.0)	204.0 (189.7–233.0)	<0.001	P1 = 0.23, P2 = <0.001, P3 = <0.001, P4 = 1.0, P5 = <0.001, P6 = 0.001, P7 = 0.11, P8 = 0.36, P9 = <0.001, P10 = <0.001
Arg	5.67 (3.17–8.14)	10.2 (6.62–15.4)	1.53 (0.0–8.54)	6.1 (0.0–14.57)	10.6 (7.65–19.5)	<0.001	P1 = <0.001, P2 = 1.0, P3 = 0.08, P4 = 0.02, P5 = 0.002, P6 = 0.25, P7 = 0.85, P8 = 0.60, P9 = 0.03, P10 = 0.24
Fisher ratio	3.53 (3.07–3.82)	1.51 (1.26–1.85)	2.29 (1.96–2.64)	1.51 (1.91–2.31)	1.92 (1.70–2.23)	<0.001	P1 = <0.001, P2 = <0.001, P3 = <0.001, P4 = <0.001, P5 = <0.001, P6 = 0.33, P7 = 0.21, P8 = 0.02, P9 = 0.38, P10 = 0.99
BTR	6.11 (5.74–7.19)	2.66 (2.02–3.50)	4.74 (3.81–5.77)	3.69 (2.83–5.20)	3.51 (3.09–4.96)	<0.001	P1 = <0.001, P2 = <0.001, P3 = <0.001, P4 = <0.001, P5 = 1.0, P6 = 0.99, P7 = 0.99, P8 = 0.07, P9 = 0.19, P10 = 1.0

P1 = control and HCC, P2 = control and HBV, P3 = control and HCV, P4 = control and coinfection, P5 = HCC and HBV, P6 = HCC and HCV, P7 = HCC and Coinfection, P8 = HBV and HCV, P9 = HBV and Coinfection, P10 = HCV and Coinfection.

## Data Availability

All data are available upon request from Samar Ebrahim Ghanem.
